# Risk factors and prognosis of recurrent wheezing in Chinese young children: a prospective cohort study

**DOI:** 10.1186/s13223-019-0351-4

**Published:** 2019-06-18

**Authors:** Jing Guo, Wenjing Zhu, Huimin Wang, Patrick G. Holt, Guicheng Zhang, Chuanhe Liu

**Affiliations:** 1 Department of Allergy, Children’s Hospital of Capital Institute of Pediatrics, Beijing, China; 2 School of Public Health, Curtin University, Perth, Australia; 3 The Curtin UWA Centre for Genetic Origins of Health and Disease, Faculty of Health and Medical Sciences, Curtin University, The University of Western Australia, Perth, Australia; 4 Telethon Kids Institute, University of Western Australia, Perth, Australia

**Keywords:** Preschool children, Wheezing, Phenotype, Risk factor

## Abstract

**Background:**

Nearly all the investigations into the risk factors for wheezing and asthma were conducted in developed countries with a high prevalence rate of asthma and allergy, but the studies in developing countries are limited. In this study, we aimed to investigate the risk factors for different wheezing phenotypes in Chinese young children and to explore the prognosis of recurrent wheezing.

**Methods:**

This cohort study contained the recruitment stage and the follow-up stage conducted by phone questionnaire survey. According to the information collected at the follow-up for wheezing episodes and remission age, our cohort was divided into transient wheezing, persistent wheezing and late-onset wheezing. The wheezing symptoms and potential risk factors were compared between these three wheezing groups.

**Results:**

From the initial 109 participants, 78.0% completed the follow-up survey. The frequency of current wheezing at followup was significantly reduced in all three groups compared to the recruitment stage (*p *< 0.01). We observe a trend that the rhinovirus (RV) and respiratory syncytial virus (RSV) infection rates were higher in the persistent wheezing group, and the overall infection rates appear to be the lowest in late-onset wheezing group at recruitment. At follow-up stage, the rates of rhinitis ever and current rhinitis were both higher in the persistent wheezing (63.0%, 50.0%) and late-onset wheezing groups (88.2%, 58.8%), compared to the transient wheezing group (14.3%, 14.3%). The incidence of current wheezing episodes increased cumulatively if the participant had concomitant risk factors of rhinitis ever, aeroallergens sensitization at recruitment, either alone or together with previous RV infection at the time of recruitment.

**Conclusion:**

While the incidence of wheezing declined overall with age, but in addition to transient wheezers, additional subsets of children manifest persistent wheeze or late onset wheeze, and moreover the risk factors for wheezing display phenotypic variability between these subgroups. Rhinitis ever and aeroallergens sensitization, either alone or together with previous RV infection, were the most significant predictors for persistent wheezing in children in an eastern environment, such as in China.

## Background

Recurrent wheezing among infant/preschoolers is very prevalent. This not only has an impact on the affected children and their families but also on the society due to increased utilization of ED visits and hospitalizations [1]. Although many young wheezers outgrow their symptoms, a significant proportion of these young children will keep wheezing throughout school age years, or even into adulthood [2]. Studies have shown that the years at preschool are crucial for asthma development as lungs and immune system develop and mature functionally [3, 4]. However, it is very difficult to identify which children with wheezing symptoms will develop asthma in later years. Early identification of recurrent wheezing in children could help physicians to improve the secondary preventive measures and suitable treatments.

To date, multiple risk factors have been identified that contribute to the development of persistent wheezing and susceptibility to asthma. These are: a family history of asthma or atopy, childhood eczema, allergic rhinitis, allergic sensitization (skin prick test and specific IgE), and early life infection with viruses [5, 6]. Notably, viral respiratory infections have been strongly associated with wheezing and susceptibility to asthma. Especially human rhinoviruses (RVs) are gaining recognition as an important risk factor for wheezing and asthma [7]. Jackson et al. found that wheezing RV illness in infancy is the most significant predictor of the development of pre-school wheezing at the age of 3 years, and also a predictor of the development of asthma at age of 6 years [8, 9]. Another recent study showed that sensitization, eczema, and RV infection are predictors for atopic asthma at the school age [10].

Nearly all the investigations into the risk factors for wheezing and asthma were conducted in developed countries with a high prevalence rate of asthma and allergy [11]. In contrast, there have been limited studies in developing countries. Although the prevalence of childhood asthma and allergy is increasing in developing countries, like China, it is still relatively low compared to that of the Western developed countries, like Australia, the UK, etc. [11, 12]. We hypothesize that the risk profiles for the prognosis of recurrent wheezing are different in developing countries, compared to developed countries. In this study, we aimed to investigate the risk factors for different wheezing phenotypes in Chinese young children and to explore the prognosis of recurrent wheezing.

## Methods

### Study design

#### Recruitment

**Fig. 1:**
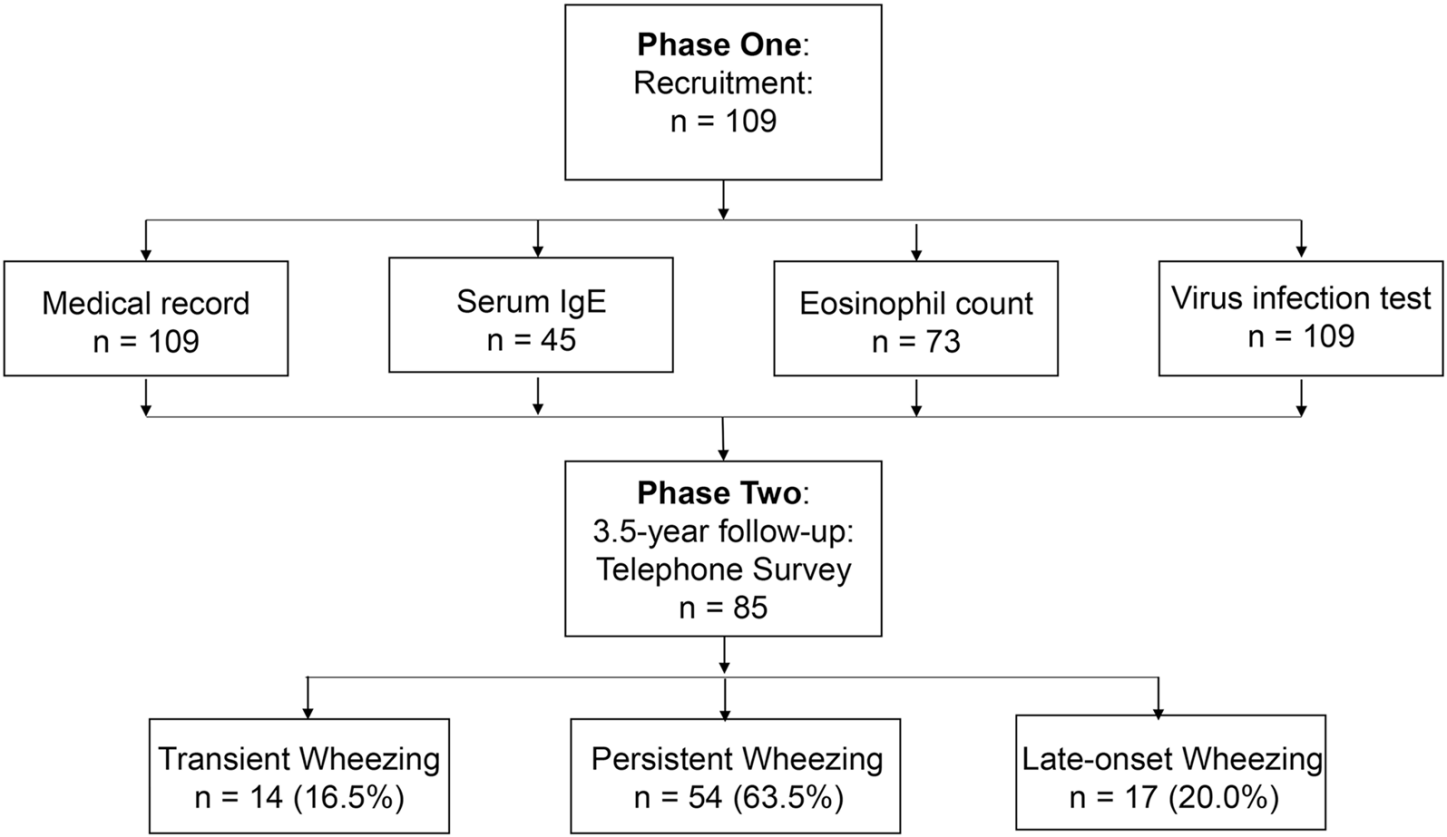
The flow chat of the study design: a total number of 109 participant were recruited at phase 1, with all of them having medical record, and virus infection test, but 45 and 73 of them had got serum IgE and EOS count tested. 85 of the participants had finished the telephone survey at follow-up stage

This longitudinal cohort study was undertaken at the Children’s Hospital of the Capital Institute of Pediatrics, Beijing, China. Figure 1 shows the flow of the study. During phase 1 (Oct 2013–May 2014) a total of 109 participants were recruited at the asthma outpatient clinic during their wheezing exacerbation. The criteria of recruitment were aged from 6 months and up to 6 years old with 3 or more episodes of doctor-diagnosed wheezing. The exclusion criteria were: history of congenital pulmonary airway malformation, bronchopulmonary dysplasia, trachea cannula, severe pneumonia, severe immunodeficiency disease, and cardiovascular disease. The parents/guardians were asked to fill in a questionnaire for their children during the recruitment. Blood was taken to measure serum total and specific IgE (sIgE) and eosinophil (EOS) count in peripheral blood. Nasopharyngeal aspirate (NPA) specimens were collected to detect virus infection, which included the detection of RV, Human Metapneumovirus (hMPV), Bocavirus, respiratory syncytial virus (RSV), parainfluenza viruses type I, II and III (PIV), influenza types A and B (Flu).

#### Follow-up stage

Phase 2 (Oct–Nov 2017) was the follow-up of the phase 1 cohort. A specialist physician at the asthma clinic conducted a telephone questionnaire survey on the child’s current wheezing status, etc.

This study was approved by the Ethics Committee of the Capital Institute of Pediatrics (SHERLL-2013072). All parents of the recruited children gave informed consent.

#### Questionnaire survey

The questionnaire at phase 1 captured baseline information on the child’s date of birth, gender, birth history, siblings, breastfed history, pets ownership (at birth, and current), mother and other family members smoking history, onset age of wheezing, past-year wheezing episodes, rhinitis history, eczema history and parental history of asthma.

The telephone questionnaire during phase 2 recorded the following items, [1] current wheezing: Wheezing episodes in the past 12 months, [2] history of doctor-diagnosed rhinitis and eczema after recruitment, and [3] current rhinitis and eczema: Symptoms of doctor-diagnosed rhinitis and eczema in the past 12 months.

### Serum IgE and peripheral blood eosinophil count

Serum was isolated for total IgE and specific IgE (sIgE) measurements using the ImmunoCap™ assays (Phadia, Sweden) on Phadia^®^ 250 System (Thermo Fisher Scientific). Any sensitization was defined as positive sIgE results against common food allergens FX5 (egg white, cow’s milk, codfish, wheat, peanut, soybean), and aeroallergens: Phad, mould mix (MX1), and house dust mite (HDM: Der p, Der f) with cutoff level of 0.35 kU/L [10]. Peripheral blood eosinophil count was obtained from XN-1000™ Hematology Analyzer (Sysmex Corporation).

### Virus infection detection

The NPA specimens were processed within 3 h after collection. RV, hMPV and Bocavirus were screened by a reverse transcription-polymerase chain reaction. Direct detection of viral antigens by fluoroimmunoassay was carried out using UItraTMDFA Respiratory Virus Screening & ID Kit (Diagnostic Hybrids, USA) for RSV, PIV type I, II and III, Flu types A and B.

### Grouping strategy

After the telephone survey of phase 2, our participants were assigned into 3 groups according to the study of Martinez et al. [5]:

1. Transient wheezing group: wheezing symptom started and remitted before age 3.
2. Persistent wheezing group: wheezing symptom started before age 3-year and persisted until age 6 or after.
3. Late-onset wheezing group: wheezing symptom started after age 3.

### Statistical analysis

Results of the continuous variables (normal distribution) were expressed as mean ± SD. Non-normal distribution data were expressed as the median and inter-quartile range. The Mann–Whitney U test was utilized to compare the past-year wheezing episodes between recruitment and follow-up stages and the Kruskal–Wallis H test was used for comparing the current wheezing episodes and the onset age of wheezing between the three wheezing phenotype groups. Variance analysis was utilized for the three groups’ comparison of EOS and logarithmic transformation of total serum IgE. Categorical variables were compared using *χ*^2^ test. If the expected values in any of the cells of the contingency table were below 5, Fisher’s exact test was used. Poisson regression was used to determine the incidence rate ratio (IRR) of current wheezing episodes at the follow up and its association with concomitant risk factors adjusted for the confounders. SPSS 17.0 was used for the statistical analysis and RStudio (Version 1.0.153) was used for creating figures.

## Results

### Characteristics of the participants and the wheezing status comparison

The average age of the 109 participants at the start of the study (phase 1) was 2.6 ± 2.3 year and 80 (73.4%) were males. At phase 2, 85 (78.0%) of the participants’ parents completed the telephone survey, and 66 (77.6%) of these children were boys. Forty-three (50.6%) of the participants were initially recruited in spring, while 24 (28.2%) and 18 (21.2%) were recruited in autumn and winter, respectively. The demographic information are shown in Table 1. There was no significant difference in the gender frequency, birth history, recruitment season, siblings, breastfed history, childcare, pet ownership, mother and other family member smoking history between the different wheezing phenotypes.

**Table 1:** Patients’ characteristics and wheezing episode

	Transient wheezing (n = 14)	Persistent wheezing (n = 54)	Late-onset wheezing (n = 17)	*χ* ^2^	*p*
Age at recruitment (mean ± SD)	1.32 ± 0.62	2.61 ± 1.44	4.57 ± 0.79	–	–
Age at follow-up (mean ± SD)	4.95 ± 0.70	6.28 ± 1.26	8.19 ± 0.75	–	–
Gender [n (%)]				0.62	0.734
Male	11 (78.6)	43 (79.6)	12 (70.6)		
Female	3 (21.4)	11 (20.4)	5 (29.4)		
Birth history [n (%)]^a^				3.60	0.134
Full-term	11 (78.6)	47 (87.0)	17 (100.0)		
Preterm	3 (21.4)	7 (13.0)	0 (0.0)		
Recruitment season [n (%)]^a^				1.24	0.891
Spring	9 (64.3)	26 (48.1)	8 (47.1)		
Autumn	3 (21.4)	16 (29.6)	5 (29.4)		
Winter	2 (14.3)	12 (22.2)	4 (23.5)		
Siblings [n (%)]^a^					
No-siblings	8 (57.1)	37 (68.5)	14 (82.4)	2.32	0.321
Breastfed history [n (%)]^a^					
Yes	14 (100.0)	50 (92.6)	15 (88.2)	1.39	0.496
Childcare [n (%)]^a^					
Yes	0 (0.0)	1 (1.9)	1 (5.9)	1.54	0.605
Pets kept when the child was born [n (%)]^a^					
Yes	2 (14.3)	8 (14.8)	2 (11.8)	0.17	1.000
Current pet ownership [n (%)]^a^					
Yes	2 (14.3)	6 (11.1)	2 (11.8)	0.40	0.892
Mother smoking history [n (%)]^a^					
Yes	1 (7.1)	1 (1.9)	1 (5.9)	2.14	0.300
Other family member smoking history [n (%)]^a^					
Yes	9 (64.3)	31 (57.4)	10 (58.8)	0.25	0.949
Onset age of wheezing (months)^b^	6.0 (5.8, 12.0)	12.0 (6.0, 24.0)	36.0 (36.0, 48.0)	43.66	*< 0.001*
Current wheezing episode at recruitment^b^	4 (3, 4)	4 (3, 5)	4 (2, 6)	0.16	0.925
Current wheezing episode at follow-up^b^	0 (0, 0)^c^	0 (0, 1)	0 (0, 1)	0.98	0.323

Italic values indicate the significance of *p* value (*p* < 0.05)

^a^Fisher’s exact test was used for groups’ comparison

^b^Variables were expressed as median (inter-quartile)

^c^Children in Transient wheezing group stopped wheezing symptom at follow-up

**Fig. 2:**
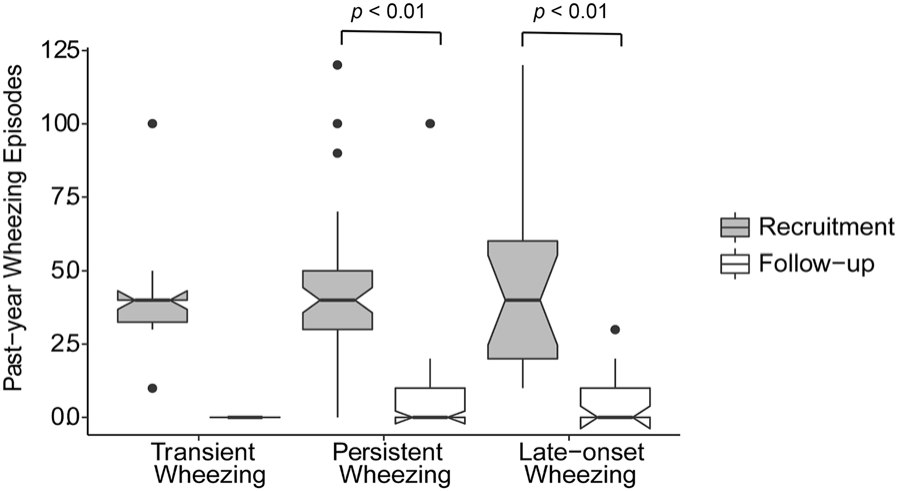
The current wheezing episodes comparison between recruitment and follow-up stages in three different phenotypes: Notched boxes showed the inter quartile ranges, lines inside the boxes represented medians, and dots were outliers

The number of current wheezing episodes had decreased in all groups, including those with persistent and late onset wheezing (both of the *p* < 0.01) (Fig. 2). However, there was no significant difference in current wheezing between the different phenotypes of wheezing groups, both at recruitment (*p *= 0.925) and the follow-up stage (*p* = 0.323) (Table 1). Comparison of the onset age of wheezing showed that the transient wheezing group started earlier than the persistent wheezing group (*p* = 0.039).

### Risk factors for the three different wheezing phenotypes

We have compared the risk factors between the three different wheezing phenotypes (Table 2). The total virus infection rates were 42.9%, 51.9%, and 29.4% for the transient, persistent, and late-onset wheezing groups, respectively. The RV infection rate was the highest among the tested viruses in all three groups, followed by the RSV positive rate. Although there was no significant difference between the three wheezing phenotype groups, we did observe a trend that the RV and RSV infection rates were higher in the persistent wheezing group, and the overall infection rate appear the lowest in late-onset wheezing group.

**Table 2:** The comparison of wheezing risk factors between the three wheezing phenotypes

	Transient wheezing (n = 14)	Persistent wheezing (n = 54)	Late-onset wheezing (n = 17)	Statistics	*p*
*Information from recruitment*					
Total virus positive [n (%)]	6 (42.9)	28 (51.9)	5 (29.4)	*χ*^2^= 2.68	0.281
RV positive [n (%)]^a^	2 (14.3)	20 (37.0)	3 (17.6)	*χ*^2^= 3.85	0.150
RSV positive [n (%)]^a^	1 (7.1)	8 (14.8)	1 (5.9)	*χ*^2^= 0.91	0.703
PIV positive [n (%)]^a^	2 (14.3)	3 (5.6)	0 (0.0)	*χ*^2^= 2.45	0.239
hMPV positive [n (%)]^a^	0 (0.0)	0 (0.0)	1 (5.9)	*χ*^2^= 3.43	0.365
Bocavirus positive [n (%)]^a^	0 (0.0)	1 (1.9)	0 (0.0)	*χ*^2^= 1.12	1.000
Flu positive [n (%)]^a^	1 (7.1)	0 (0.0)	0 (0.0)	*χ*^2^= 3.82	0.165
Serum IgE test (n = 45)	n = 7	n = 29	n = 9		
Total IgE (ku/L)	59.8 ± 35.1	193.7 ± 206.4	237.7 ± 202.7	–	–
Log transformed total IgE	0.7 ± 0.4	2.1 ± 0.4	2.3 ± 0.3	*F *= 5.03	*0.011*
Atopy	4 (57.1)	24 (82.8)	9 (100.0)	*F *= 4.36	0.076
Aeroallergens positive (Phad) [n (%)]^a^	1 (14.3)	11 (37.9)	7 (77.8)	*χ*^2^= 6.71	*0.031*
Mould mix positive (MX1) (n = 38)^a^	1 (16.7)	6 (25.0)	5 (62.5)	*χ*^2^= 4.16	0.128
House dust mite positive (HDM) (n = 37)^a^	0 (0.0)	7 (30.4)	4 (50.0)	*χ*^2^= 3.88	0.167
Food allergens positive [n (%)]^a^	2 (28.6)	15 (51.7)	5 (55.6)	*χ*^2^= 1.39	0.623
EOS in peripheral blood (n = 68)	n = 10	n = 44	n = 14		
Percentage (%)	2.60 ± 2.54	3.65 ± 2.41	5.06 ± 2.27	*F *= 3.24	*0.045*
Absolute count (× 10^9^/L)	0.27 ± 0.24	0.39 ± 0.30	0.49 ± 0.30	*F *= 2.56	0.085
Parental asthma history [n (%)]^a^	1 (7.1)	5 (9.3)	1 (5.9)	*χ*^2^ = 0.23	1.000
*Information from follow-up*					
Rhinitis ever [n (%)]	2 (14.3)	34 (63.0)	15 (88.2)	*χ*^2^ = 18.00	*0.000*
Current rhinitis [n (%)]	2 (14.3)	27 (50.0)	10 (58.8)	*χ*^2^ = 7.14	*0.028*
Eczema ever [n (%)]	8 (57.1)	36 (66.7)	10 (58.8)	*χ*^2^ = 0.64	0.727
Current eczema [n (%)]^a^	1 (7.1)	7 (13.0)	3 (17.6)	*χ*^2^ = 0.74	0.804

Italic values indicate the significance of *p* value (*p* < 0.05)

^a^Fisher’s exact test was used for groups’ comparison

There was a significant difference for ‘rhinitis ever’ between the three groups (*p* < 0.01). The rhinitis rates of the persistent and late-onset wheezing groups were both higher than the transit wheezing group (both *p* < 0.01). We found that the current rhinitis rate of the persistent wheezing and late-onset wheezing groups was also higher than transient wheezing group at the follow-up stage (*p* = 0.016, *p* = 0.011).

The total IgE of the transient wheezing group was significantly lower than the other two groups (*p* < 0.05). However, there was no significant difference of total IgE level between the persistent and late-onset wheezing groups. The prevalence of a positive specific IgE to aeroallergen was highest in the late-onset wheezing group, but no difference was observed in the prevalence of mould mix, house dust mite (HDM) and food allergen sIgE positive. Significant difference was detected for the percentage of EOS in peripheral blood (*p* = 0.045) between the three wheezing groups, with the highest EOS percentage in late-onset wheezing group.

### The concomitant risk factors for the incidence of current wheezing episodes

**Fig. 3:**
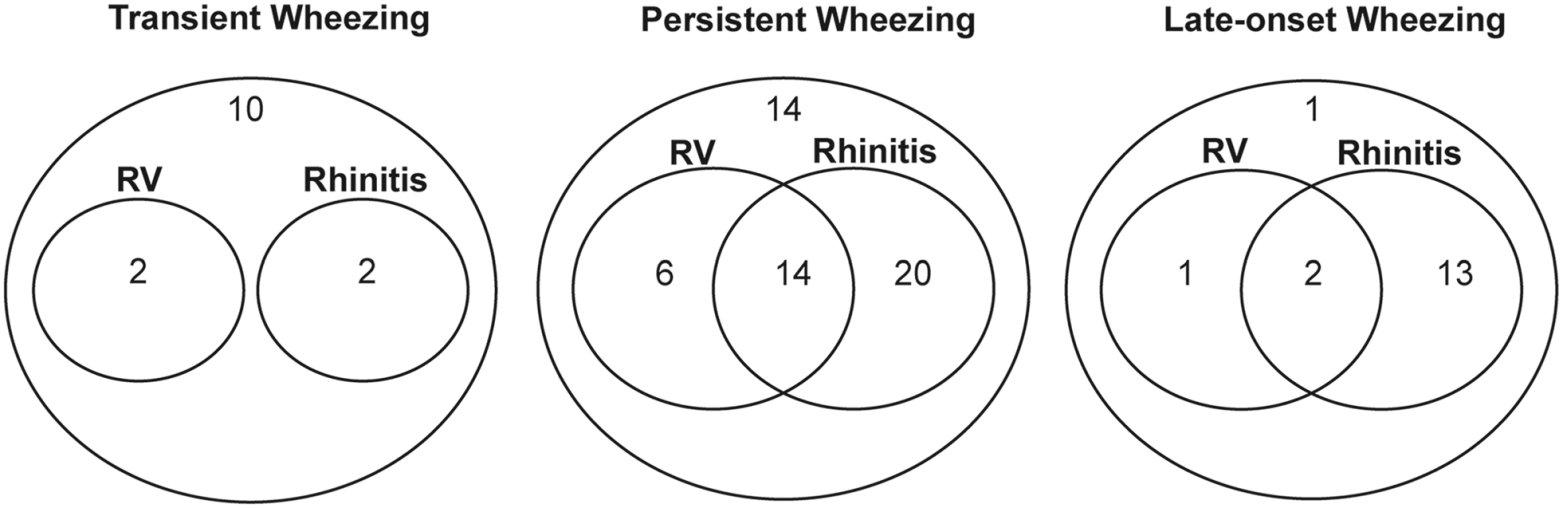
Patient numbers of wheezing phenotypes with sole and overlapping risk factors (RV and rhinitis): Venn diagrams were draw to show the concomitant of RV infection and rhinitis in the three different wheezing phenotypes

The incidence of current wheezing episodes increased cumulatively if the participant had concomitant risk factors (Table 3). The significance did not change after adjustment for the confounders we investigated in this study, including gender, birth history, number of siblings, breastfed history, childcare, pets ownership, mother and other family members’ smoking history, onset wheezing age (months), past-year wheezing episode at recruitment and parental asthma history. Four confounders were finally included in the regression model. The incidence rate ratios (IRRs) of wheezing increased from with either rhinitis or RV infection (3.61, *p* < 0.01) to both (4.54, *p* = 0.01), compared to neither, respectively. The IRRs were also significantly high with either aeroallergens positive or RV infection (2.54, *p* = 0.05), and marginally significant with both (3.44, *p* = 0.07). In addition, children with both rhinitis and aeroallergens positive were 4.36-fold higher of IRR than children with none of the risk factors (*p* < 0.01). Poisson regression showed concomitant rhinitis and/or RV infection strongly indicated an increased risk of wheezing at the follow up and Fig. 3 visualizes the overlapping characteristics of these two risk factors among the three different wheezing phenotypes. The overlap of rhinitis and RV infection was 14 (25.9%) in persistent wheezing group compared to 0 (0.0%) and 2 (11.8%) in the transient and late-onset wheezing groups, respectively.

**Table 3:** The incidence rate ratios (IRRs) of concomitant risk factors for the incidence of current wheezing episodes

	IRRs	95% CI		*p*
		Lower	Upper	
No rhinitis and no RV	–	–	–	–
Rhinitis or RV	3.61	1.46	8.94	*0.005*
Rhinitis and RV	4.54	1.43	14.46	*0.010*
No eczema and no RV	–	–	–	–
Eczema or RV	1.50	0.69	3.25	0.307
Eczema and RV	2.17	0.76	6.19	0.149
No aeroallergens positive and no RV	–	–	–	–
Aeroallergens positive or RV	2.54	1.00	6.45	*0.050*
Aeroallergens positive and RV	3.44	0.89	13.29	0.073
No rhinitis and no aeroallergens positive	–	–	–	–
Rhinitis or aeroallergens positive	2.25	0.86	5.87	0.097
Rhinitis and aeroallergens positive	4.36	1.56	12.14	*0.005*

Italic values indicate the significance of *p* value (*p* < 0.05)

Poisson regression model was employed after adjusting for gender, onset wheezing age (months), past-year wheezing episode at recruitment and parental asthma history

*95% CI* 95% confidence intervals

## Discussion

The prevalence of wheezing has been increasing recently in China [13] but the prognosis of recurrent wheezing during young age is unknown in developing countries. To the best of our knowledge, this is the first longitudinal study conducted in China that investigates the prognosis of recurrent wheezing in young children and identifies risk factors for different wheezing phenotypes.

The strength of our study is that it includes the comprehensive collection of medical records, viral etiology, serum IgE test, and EOS count at study entry, and is combined with a 3.5-year follow-up by an experienced asthma clinic specialist practitioner. We found that rhinitis and aeroallergen sensitization, either alone or together with early RV infection, were the most significant predictors for subsequent persistent and late onset wheeze in China. Previously, an observational study conducted in Turkey investigated the short-term prognosis and risk factors of recurrent wheezing in the first 3 years of life [14]. Maternal smoking during pregnancy and emergency room admissions were significant risk factors for the persistence of wheezing. A study in Mozambique by O’Callaghan-Gordo et al. showed that an initial episode of lower respiratory infection (LRI) with RV during infancy could increase the risk of wheezing [15].

We found that the number of wheezing episodes had decreased significantly during the follow-up stage and the onset age of wheezing was earlier in the transient wheezing than in the persistent wheezing group. The findings are consistent with earlier studies from Martinez et al. [5] and Matricardi et al. [16] showing that the incidence of wheezing declined with age. The respiratory system gradually undergoes development with age which may be an important reason for the decrease in the number of wheezing episodes [17]. In addition, our findings echo the previous reports that the risk factors of wheezing also show phenotypic variability [18, 19]. A large population-based cohort in the UK showed that episodic viral wheeze decreased with age, but multiple trigger wheeze (mainly due to exercise or aeroallergen-associated wheeze) increased [20]. Martinez suggested that transient wheezing in young children is not associated with an atopic predisposition [21].

It is well known that respiratory tract virus infection is an important cause for early childhood wheezing and asthma [22]. Jackson et al. revealed that RV and RSV are the main viruses that cause wheezing, and RV infection can affect the long-term prognosis of children with wheezing [23]. Early life RV-induced wheezing can increase the asthma risk at school age [24]. We observed 37% of RV in children with persistent wheezing and less than 20% of RV in children with the other wheezing phenotypes, although these differences were not statistically different due to the relatively small sample size. To date, studies on the mechanisms of respiratory virus-induced wheeze in children has surged, driven by efforts to find better interventions to reduce wheezing exacerbations. A recent review recapitulated that RV type C was associated with a decrease in expression of a cellular receptor specific for this virus-CDHR3, and a decrease in interferon-β expression [25]. The shift to focus on the mechanisms and new intervention methods is promising.

Allergic rhinitis (AR) is often associated with asthma and asthma is present in 15% to 38% of the patients with AR, and nasal symptoms are found in 6% to 85% patients with asthma [26]. Indeed we found that the incidence of rhinitis ever and current rhinitis were more common in the persistent and late-onset wheezing groups compared to the transient wheezing group. The sIgE results showed that a positive allergy response to aeroallergens was lower in the transient wheezing groups. Serum IgE levels has long been associated with asthma and allergic sensitization is an independent predictor for the persistence of wheezing [27, 28]. Interestingly, when we analyzed the concomitant risk factors we found that the increase of wheezing episodes in the year preceding follow up was associated with overlapping RV and/or rhinitis, RV and/or aeroallergens sensitization, rhinitis and aeroallergens sensitization. This confirms RV, aeroallergens and rhinitis, more likely working together are dominant risk factors for persistent wheezing in China. As noted above, this constellation of risk factors closely resembles those reported for developed countries, despite the fact that as a developing country China has different social/economic contexts and lower allergy prevalence compared to industrialized countries.

In this regard, a community-based birth cohort study demonstrated that viral infections interact with atopic sensitization in infancy to enhance the susceptibility to asthma development and the occurrence of both of the two factors is associated with maximal risk for subsequent asthma [29]. Jackson and colleagues also showed that asthma rate was highest in infants with both sensitization and RV-related wheezing, but RV-related wheezing was the most significant predictor of subsequent asthma in the Childhood Origins of ASThma (COAST) study [8]. Consistently, a recent cohort study revealed that first RV-induced wheezing alone or together with sensitization could predict atopic school-aged asthma [10]. Notably, in our result, children in persistent and late-onset wheezing groups are approximately equivalently “atopic” relative to the children in transient wheezing group. We speculate that the reason why onset of asthma is faster in the persistent wheezing group is because the early impact of viral infections on them was higher, whereas it took longer time for the late-onset wheezing group to accumulate enough viral-associated tissue damage to catch up and start expressing symptoms. On the other hand, we suggest that the late-onset wheezing group could be driven by atopy alone and in the absence of high level viral infection, it takes longer for lung function to deteriorate. However, the wheezing symptom in transient wheezing group is simple and mainly due to the congenitally smaller airways not being able to manage viral inflammation and children in this group grow out of that because they don’t have the “second hit” from atopy.

This study has limitations that should be taken into account. Firstly, we acknowledge the small sample size of this study. Nonetheless, the conclusion should be interpreted with caution. Secondly, not all the participants had serum IgE and EOS count measured at recruitment. This is because that not all patients were covered by health insurance for serum IgE test due to policy (e.g. outpatient clinic attendance in non-local tertiary hospitals). However, this novel Chinese cohort provided insights into the risk factors and prognosis of early life wheezing in an eastern environment. The focus for future researches should be larger, preferably multicentre, study cohorts with extensive follow-up to elucidate the risk factors in the development of asthma in children living in developing countries.

In conclusion, we show that while the incidence of wheezing declined overall with age, but in addition to transient wheezers, additional subsets of children manifest persistent wheeze or late onset wheeze, and moreover the risk factors for wheezing display phenotypic variability between these subgroups. Rhinitis ever and aeroallergens sensitization, either alone or together with previous RV infection, were the most significant predictors for persistent wheezing in children in China.

## Data Availability

The data that support the findings of this study are not publicly available due to the undergoing of more analysis, but are available from the corresponding author upon reasonable request.

## Abbreviations

RV: rhinovirus
RSV: respiratory syncytial virus
hMPV: human metapneumovirus
PIV: parainfluenza virus
Flu: influenza
EOS: eosinophil
IRR: incidence rate ratio
HDM: house dust mite
LRI: lower respiratory infection
AR: allergic rhinitis
